# 2-Hydroxyestradiol Overcomes Mesenchymal Stem Cells-Mediated Platinum Chemoresistance in Ovarian Cancer Cells in an ERK-Independent Fashion

**DOI:** 10.3390/molecules27030804

**Published:** 2022-01-26

**Authors:** Hazem Khamaisi, Hatem Mahmoud, Jamal Mahajna

**Affiliations:** 1 Department of Nutrition and Natural Products, Migal—Galilee Research Institute, Kiryat Shmona 11016, Israel; hazemkh@migal.org.il (H.K.); hatem.gu13@gmail.com (H.M.); 2 Department of Biotechnology, Tel-Hai College, Kiryat Shmona 11016, Israel

**Keywords:** ovarian cancer, drug resistance, ERK, 2HE2, platinum

## Abstract

Ovarian cancer (OC) is the second most common type of gynecological malignancy. Platinum (Pt)-based chemotherapy is the standard of care for OC, but toxicity and acquired chemoresistance has proven challenging. Recently, we reported that sensitivity to platinum was significantly reduced in a co-culture of OC cells with MSC. To discover compounds capable of restoring platinum sensitivity, we screened a number of candidates and monitored ability to induce PARP cleavage. Moreover, we monitored platinum uptake and expression of ABC transporters in OC cells. Our results showed that 2-hydroxyestradiol (2HE2), a metabolite of estradiol, and dasatinib, an Abl/Src kinase inhibitor, were significantly effective in overcoming MSC-mediated platinum drug resistance. Dasatinib activity was dependent on ERK1/2 activation, whereas 2HE2 was independent of the activation of ERK1/2. MSC-mediated platinum drug resistance was accompanied by reduced intracellular platinum concentrations in OC cells. Moreover, MSC co-cultured with OC cells resulted in downregulation of the expression of cellular transporters required for platinum uptake and efflux. Exposure to 2HE2 and other modulators resulted in an increase in intracellular platinum concentrations. Thus, 2HE2 and dasatinib might act as sensitizers to restore platinum drug sensitivity to OC cells and thus to limit TME-mediated chemoresistance in OC.

## 1. Introduction

Chemoresistance is frequently encountered in ovarian cancer (OC) patients treated with platinum-based chemotherapy. The mechanisms that underlie OC platinum chemoresistance are largely unknown. However, platinum chemoresistance might result from decreased platinum accumulation within the cancer cells, elevated drug inactivation by metallothionein and glutathione, and enhanced DNA-repair activity [1,2]. In addition to chemoresistance resulting from alteration within the cancer cells [3,4], an increasing number of studies have also implicated tumor microenvironments (TME) in cancer chemoresistance [5,6].

OC ranks as the second most common type of gynecological malignancy and has poor survival rates [7]. Platinum-based chemotherapy represents the standard of care for OC. However, toxicity and acquired resistance have proven challenging in the treatment of patients with OC [8,9]. In OC, more than one-third of cancer patients present with malignant ascites at initial diagnosis. Malignant ascites acts as a reservoir for a complex mixture of metabolites, soluble factors, and cellular compartments, providing a pro-inflammatory and tumor-promoting microenvironment for the OC cells that could also be associated with chemoresistance [6,10,11,12,13,14]. Previously, we demonstrated that direct co-culture of OC with mesenchymal stem cells (MSC) conferred chemoresistance to therapeutic agents including paclitaxel, colchicine, and platinum compounds, accompanied by blocking of ERK1/2 activation [15]. Additionally, we demonstrated that the combination of a platinum drug with fisetin and other flavonoids restored platinum drug sensitivity to OC cells co-cultured with MSC accompanied by re-activation of ERK1/2 [15].

Reduced intracellular platinum accumulation has been consistently shown to correlate with resistance in tumors. Platinum intracellular concentration is regulated by the copper transporters CTR1, CTR2, ATP7A, and ATP7B that are essential for uptake and efflux of platinum into cells [16,17,18]. 

Our findings that chemoresistance is promoted by direct co-culture of MSC with OC cells were not limited to platinum compounds but included other chemotherapeutics such as paclitaxel and colchicine [15], arguing that other transporters such as ABC transporters might be involved. 

ABC transporters consist of 49 transporter proteins that are classified into seven subfamilies, ABCA to ABCG. The subfamilies are located in the cell membrane and have diverse functions including protecting cells from some anti-cancer drugs [19]. Higher expressions of these transporters have actively participated in multidrug resistance (MDR) of conventional chemotherapeutics such as doxorubicin (Dox), and other targeted therapies such as imatinib [20]. 

Cancer chemoresistance is also mediated by the activity of cellular signaling molecules, thus we aimed at identifying such relevant signaling pathways by evaluating the ability of a variety of pharmaceutical inhibitors to overcome cancer chemoresistance in our model system. 

2-methoxyestradiol (2ME2) is a natural metabolite of the endogenous estrogen hormone 17β-estradiol that is devoid of estrogenic activity [21]. Previously, 2ME2 was reported to exhibit anti-angiogenic activity by inhibiting HIF1α function [22,23], which is implicated in promoting chemoresistance in tumor cells [24]. 2-hydroxyestradiol (2HE2) is an estradiol metabolite that serves as a prodrug for 2ME2 and converts efficiently to 2ME2 [25].

Our current research findings demonstrated that chemoresistance promoted by direct co-culture of OC with MSC cells might be reversed by 2HE2 and dasatinib, similar to results previously reported with fisetin. However, OC platinum sensitizing activity of fisetin and dasatinib were found to be ERK1/2 dependent, whereas the activity of 2HE2 was ERK1/2 independent. 

## 2. Results

Previously, we synthesized a bis-octanoatoplatinum (IV) complex (RJY13), a cisplatin derivative with octanoate as an axial ligand, which exhibited a strong anti-proliferative effect on the cisplatin-resistant and cisplatin-sensitive ovarian cells [26]. Moreover, we demonstrated that direct co-culture of OC cells with mesenchymal stem cells (MSC) conferred chemoresistance to the platinum compound (RJY13) that was accompanied by blocking of ERK1/2 activation [15]. Our previous findings demonstrated that the flavonoid fisetin restored platinum drug sensitivity to OC cells co-cultured with MSC accompanied by re-activation of ERK1/2 [15]. 

### 2.1. 2-Methoxyestradiol and Dasatinib Restore Platinum Sensitivity to OC Cells in Direct Co-Culture with MSC 

**Figure 1:**
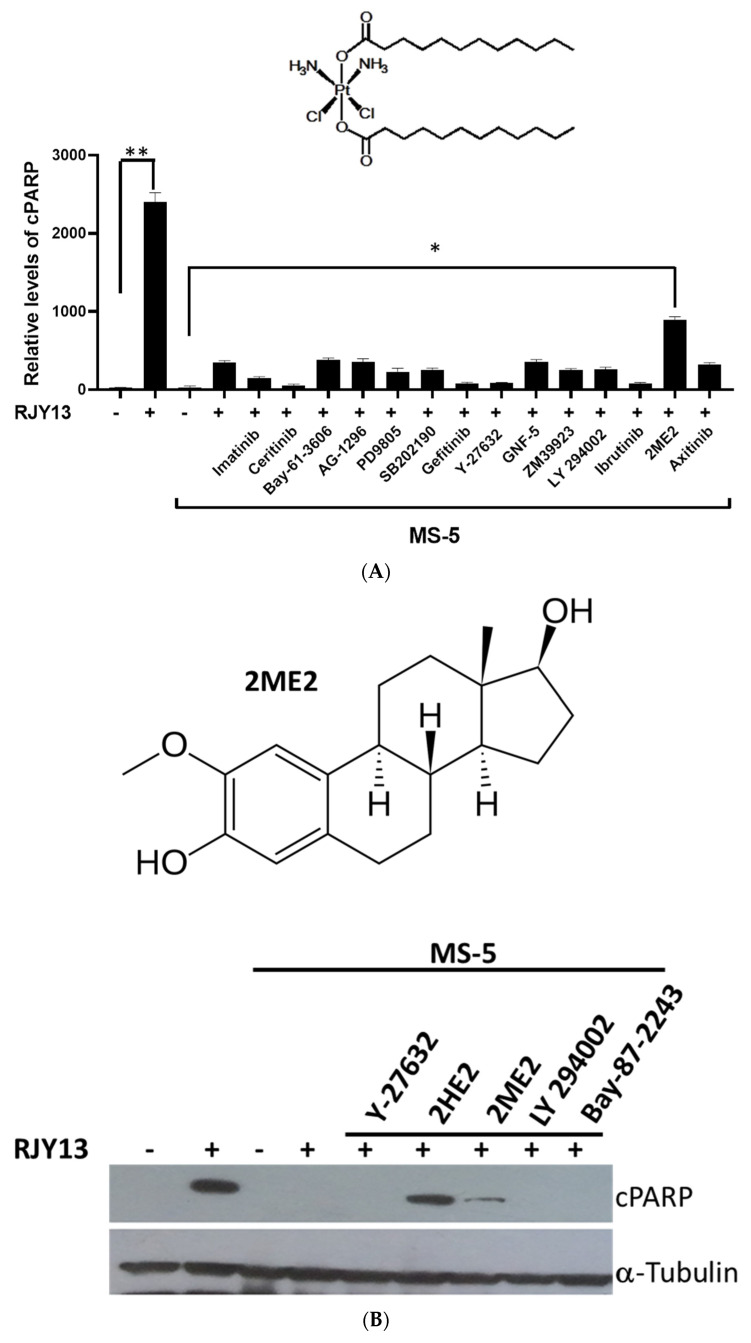

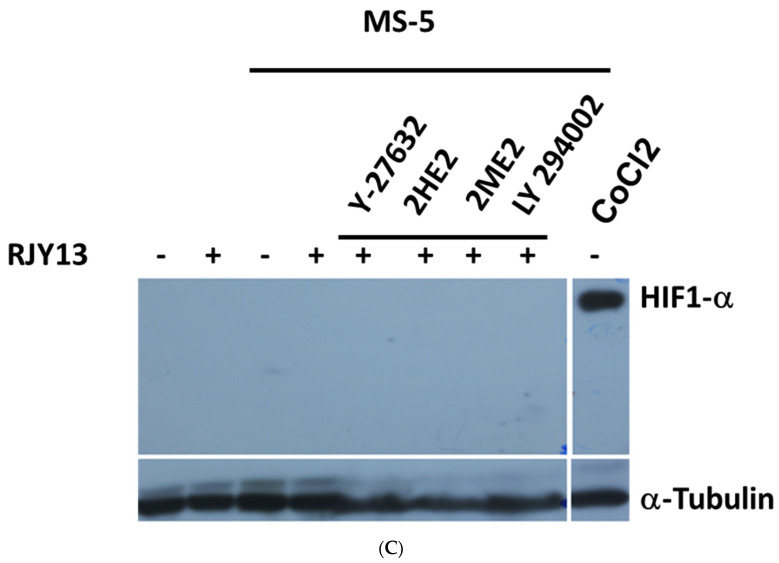
2-methoxyestradiol restores platinum drug sensitivity to A2780CisR co-cultured with MS-5. (**A**) A2780CisR cells co-cultured with MS-5 and treated with RJY13 (5 μM) and combined with 10 μM of pharmaceutical inhibitors for 24 h. Cell lysates were used to monitor levels of cleaved PARP using PARP ELISA kit [15]. Pharmaceutical inhibitors including imatinib, inhibitor of Abl kinase [34], ceritinib, inhibitor of Alk kinase [35], Bay-61-3606 inhibitor of Syk kinase [36], tyrphostin AG-1296 inhibitor of PDGFR kinase [37], PD98059 inhibitor of MEK kinase [38], SB202190 inhibitor of p38 MAPK [39], gefitinib inhibitor of EGFR kinase [40], Y-27632 ATP-competitive inhibitor of ROCK-I and ROCK-II kinases [41], GNF-5 allosteric inhibitor of Abl kinase [42], ZM39923 inhibitor of JAK1/3 kinase [43], LY 294002 broad-spectrum inhibitor of PI3K kinase [44], ibrutinib inhibitor of Btk kinase [45], 2-methoxyestradiol endogenous metabolite of 17β-estradiol [46], and axitinib inhibitor of VEGFR kinases [47]. The chemical structure of RJY13 is also shown. (**B**) A2780CisR cells were treated with RJY13 (5 μM) in monoculture or in co-culture with MS-5 in the presence of 10 μM of Y-27632, 2-hydroyestadiol, a prodrug of 2ME2, 2ME2, LY 294002, and BAY 87-2243 selective hypoxia-inducible factor-1 (HIF-1) inhibitor [30]. The chemical structure of 2ME2 is also shown. Levels of cleaved PARP were monitored by immunoblotting. (**C**) A2780CisR cells were treated with RJY13 (5 μM) in monoculture or in co-culture with MS-5 in the presence of 10 μM of Y-27632, 2-hydroyestadiol, 2ME2, LY 294002. In addition, A2780CisR cells were treated with 50 mM of CoCl_2_. α-Tubulin was used as a loading control. * *p* < 0.01 and ** *p* < 0.001. The experiment was repeated twice with comparable outcomes.

In this study, we utilized species-selective antibody-recognizing cleaved human Poly(ADP-Ribose Polymerase 1) (PARP1), but not murine cleaved PARP1, to monitor apoptosis induction in OC cells. Levels of cleaved PARP1 were monitored using ELISA or immunoblotting assays [27] in A2780CisR cells, a cisplatin-resistant OC cell line exposed to our novel prodrug RJY13 [26]. In an attempt to identify compounds that are active in restoring platinum sensitivity to OC cells directly co-cultured with MSC [15], a number of pharmaceuticals compounds were included with RJY13 and exposed to OC cells co-cultured with MS-5. The results demonstrated that almost all of the pharmaceutical tested compounds failed to restore platinum sensitivity to A2780CisR when co-cultured with MS-5, except for 2-methoxyestradiol (2ME2), which exhibited partial activity in restoring platinum sensitivity (Figure 1A). 

Results demonstrated that exposure to RJY13 caused substantial cleavage of PARP1 as indicative of apoptosis induction and direct co-culture with MS-5, blocking the ability of RJY13 to stimulate PARP1 cleavage (Figure 1B). Moreover, 2ME2 at 10 μM restored partial sensitivity to platinum in the presence of MS-5 (Figure 1B). Interestingly, 2HE2 exhibited enhanced activity compared with 2ME2. In contrast, other pharmaceutical compounds demonstrated no activity in restoring platinum sensitivity to OC cells co-cultured with MS-5. 

Since 2ME2 was reported as an anti-angiogenic modulator that affects HIF1α function [22,23], we speculated that if 2ME2 is an inhibitor of HIF1α, co-culture with MS-5 might lead to upregulation of HIF1α to promote chemoresistance [28,29]. Thus, initially, we examined the ability of MS-5, when co-cultured with OC cells, to upregulate HIF1α (by immunoblotting). No increase in HIF1α levels was observed in OC cells co-cultured with MSC (Figure 1C). Further support for the lack of involvement of HIF1α in MSC-mediated drug resistance was obtained by using selective HIF1α inhibitors such as BAY 87-2243 [30]. Our preliminary data demonstrated that exposure to BAY 87-2243 did not restore platinum sensitivity to OC cells, providing further support to the hypothesis that HIF1α may not be involved in drug resistance in OC cells when directly co-cultured with MSC (Figure 1B). 

Our previous data demonstrated that re-activation of ERK1/2 phosphorylation in direct co-culture of OC cells with MSC resulted in restoring platinum drug sensitivity to OC cells [15]. Dasatinib, an FDA-approved multi-targeted kinase inhibitor of BCR/ABL and Src kinases [31] was reported to induce ERK phosphorylation in acute myeloid leukemia (AML) [32] and during stimulated pigmentation of ex vivo cultured skin [33]. Thus, we included dasatinib in this study as an ERK activator to examine its potential for restoring platinum sensitivity to OC cells co-cultured with MSC. 

**Figure 2:**
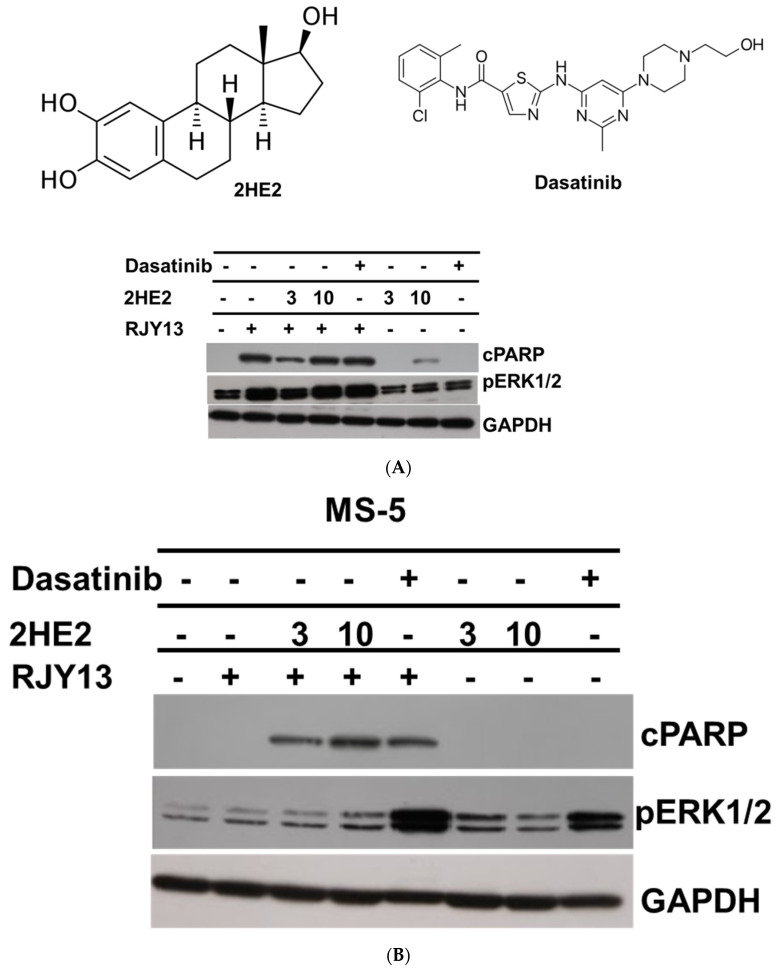

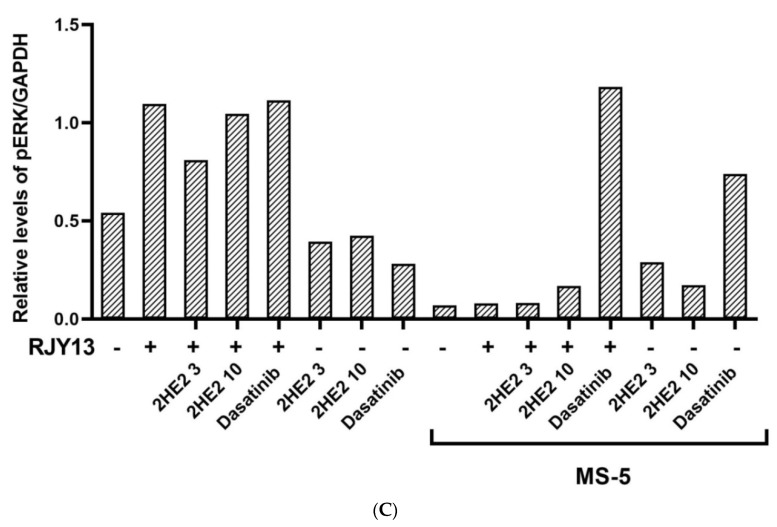
2HE2 restored platinum sensitivity to OC cells independent of phospho-ERK1/2. (**A**) A2780CisR cells were treated with 2HE2 (3 and 10 μM) and dasatinib (5 μM) alone or in combination with RJY13 (5 μM) and levels of cleaved PARP as well as phospho ERK1/2 were monitored by immunoblot. (**B**) A2780CisR cells co-cultured with MS-5 were treated with 2HE2 (3 and 10 μM) and dasatinib (5 μM) alone or in combination with RJY13 (5 μM) and levels of cleaved PARP as well as phosphoERK1/2 were monitored by immunoblot. GAPDH was used as a loading control. (**C**) pERK1/2 levels relative to levels of GAPDH were calculated and blotted. The experiment was repeated twice with comparable outcomes.

A2780CisR cells were treated with RJY13 in the presence and absence of MS-5 cells and the abilities of 2HE2 and dasatinib to restore platinum sensitivity were monitored. In addition, we monitored changes in levels of phospho-ERK1/2. Results demonstrated that exposure to RJY13 stimulated PARP cleavage and that the presence of MS-5 reduced platinum sensitivity (Figure 2). Moreover, co-exposure of 2HE2 (3 or 10 μM) restored platinum sensitivity to OC cells as evident by the presence of cleaved PARP in the immunoblot. Interestingly, exposure of OC cells to 2HE2 alone had minimal effect on apoptosis induction. Only at the highest concentration of 10 μM did we observe moderate apoptosis induction in A2780CisR in monoculture, but not in co-culture. Re-sensitization of OC cells to platinum in co-culture was observed only when RJY13 was combined with 2HE2. Exposure to RJY13 caused significant stimulation of phospho-ERK1/2. In contrast, exposure of OC cells to 2HE2 alone did not upregulate levels of phospho-ERK1/2. Moreover, in co-culture with MS-5, RJY13 failed to stimulate phospho-ERK1/2, and when combined with 2HE2 resulted only in a marginal increase in levels of pho-ERK1/2 (Figure 2). Dasatinib was active in restoring platinum sensitivity to OC cells co-cultured with MS-5. In contrast to 2HE2, exposure to dasatinib upregulated phospho-ERK1/2 levels in co-culture of OC cells, but not in mono-culture of OC cells. This finding suggests that restoration of platinum sensitivity might be ERK1/2-dependent [15] (flavonoids and dasatinib) or an independent phenomenon (2HE2). 

### 2.2. The Role of Activated ERK in Restoring Platinum Sensitivity to OC Cells 

**Figure 3:**
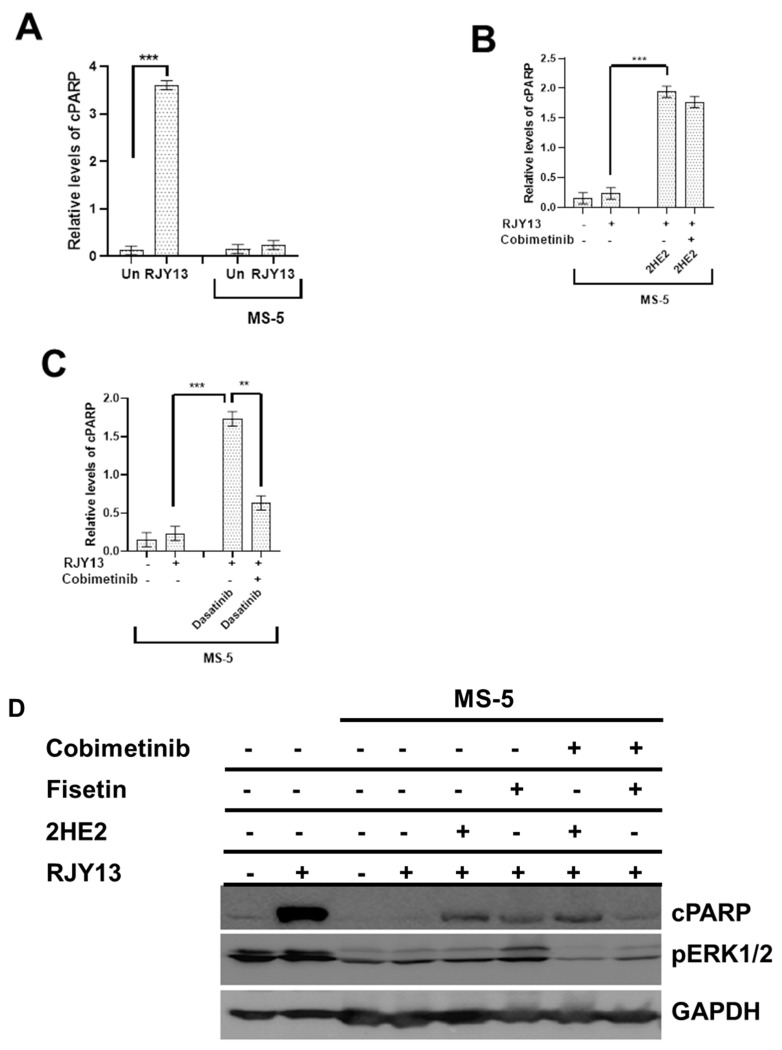
The role of ERK1/2 activity in mediating 2HE2 activity in restoring platinum sensitivity to OC cells. (**A**). A2780CisR cells treated with RJY13 (5 μM) grown as a monolayer or in direct co-culture with MSC-5. (**B**). A2780CisR cells treated with RJY13 (5 μM) grown in direct co-culture with MS-5 cells and exposed to 2HE2 (10 μM) alone or in combination with cobimetinib (2 μM) for 24 h. (**C**). A2780CisR cells treated with RJY13 (5 μM) grown in direct co-culture with MS-5 cells and exposed to dasatinib (5 μM) alone or in combination with cobimetinib (2 μM) for 24 h. Cell lysates in A-C were used to monitor levels of cleaved PARP using the PARP ELISA kit [15]. (**D**). Immunoblot of A2780CisR cells grown as a monolayer or in direct co-culture with MSC-5 and exposed to RJY13 (5 μM) combined with 2HE2 (10 μM) or fisetin (10 μM) alone or in combination with cobimetinib (2 μM). Filters were probed with anti-cPARP, pERK1/2, and GAPDH that was used as a loading control. ** *p* < 0.01 and *** *p* < 0.001.

To test the role of activated ERK in restoring platinum sensitivity to OC cells, we utilized cobimetinib, an orally bioavailable small-molecule inhibitor of mitogen-activated protein kinase kinase 1 (MEK1), which also inhibits ERK phosphorylation [48], and monitored its ability to affect restoration of platinum sensitivity by 2HE2, dasatinib, and fisetin. OC cells were exposed to platinum in the presence of 2HE2, dasatinib, or fisetin combined with cobimetinib in monoculture and in direct co-culture with MS-5. Results demonstrated that co-culture caused a significant reduction in the level of cleaved PARP as expected (Figure 3). Moreover, the presence of 2HE2 in direct co-culture condition partially restored platinum sensitivity to OC as evident by increased levels of cPARP (Figure 3B). The combination of cobimetinib with 2HE2 exhibited minimal effect on cPARP levels, demonstrating that restoration of platinum sensitivity to OC cells by 2HE2 is not dependent on activated ERK1/2 (Figure 3B,D). This finding supports prior results demonstrating that exposure to 2HE2 has minimal effect on ERK1/2 activation (Figure 2B). Similarly, we examined the dependency of dasatinib function in restoring platinum sensitivity to OC upon activation of ERK1/2. Results demonstrated that the combination with cobimetinib significantly reduced levels of cPARP as a result of exposure to RJY13 in the presence of dasatinib, suggesting that activity of dasatinib in restoring platinum drug sensitivity to OC cells is dependent on ERK1/2 activity (Figure 3C). In line with our previous results [15], the presence of fisetin partially restored OC sensitivity to the platinum compound, which was accompanied by reactivation of ERK (Figure 3D). Moreover, the combination with cobimetinib significantly reduced levels of cPARP as a result of exposure to RJY13 in the presence of fisetin, demonstrating that the activity of fisetin in restoring platinum drug sensitivity to OC cells is dependent on ERK1/2 activity (Figure 3D) [15]. 

### 2.3. Platinum Intracellular Concentration in OC Cells Co-Cultured with MSC

**Figure 4:**
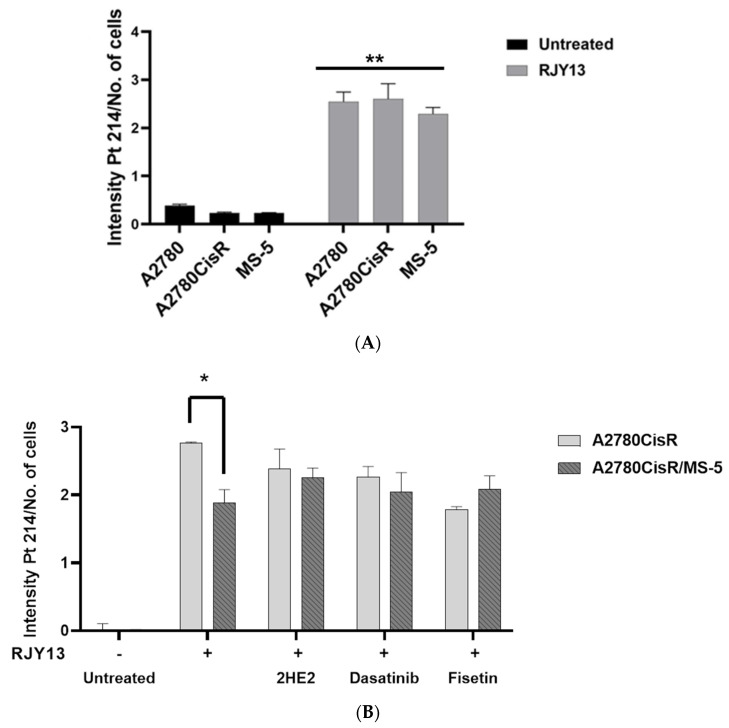
Platinum intracellular concentration in OC cells. A2780CisR, A2780, and MS-5 cells were exposed to RJY13 (5 μM) for 6 h as described in materials and methods. Cells were collected, mineralized with 65% HNO3, and then completely dried. Dry platinum-containing material was measured at *m*/*z* 214 by an ICP-OES instrument (Agilent Technologies, Santa Clara, CA, USA). (**A**). Cells growing as monolayers were exposed to RJY13 and levels of platinum were measured. (**B**). A2780cisR grown as monolayers or in direct co-culture with MS-5 were exposed to RJY13 in combination with 2HE2 (10 μM), dasatinib (5 μM), or fisetin (10 μM). * *p* < 0.05 and ** *p* < 0.01.

Levels of platinum intracellular concentration were obtained as previously published [26,49]. The different cells were exposed to RJY13 for 6 h and the intracellular level of platinum was monitored. The results demonstrated that platinum intracellular concentration (Pt 214 as well as others such as Pt203, Pt177, and Pt217) increased, on average, by 8–10-fold after exposure but was very minimal in untreated cells (Figure 4A). Next, we monitored platinum concentration in OC cells co-cultured with MS-5 and in combination with 2HE2, dasatinib, and fisetin. Results demonstrated that platinum intracellular concentration normalized to cell number was significantly increased in A2780CisR cells that were exposed to 5 μM RJY13 for 6 h (Figure 4B). Levels of platinum intracellular concentration normalized to cell number were decreased by 32% in A2780CisR cells co-cultured with MS-5 and exposed to 5 μM RJY13 for 6 h (Figure 4).

Furthermore, combination with 2HE2, dasatinib, or fisetin minimizes the decrease in platinum concentration in co-culture conditions to a 6, 10, and 17 % difference, respectively. Thus, it is reasonable to speculate that the ability of 2HE2 and other modulators to restore platinum sensitivity to OC cells in co-culture conditions might be affected by increasing the intracellular platinum concentration. 

### 2.4. Levels of Platinum Transporters in Direct OC Co-Cultured with MS-5

Platinum intracellular concentration is regulated by the copper transporters CTR1, CTR2, ATP7A, and ATP7B, which are essential for platinum compounds’ uptake and efflux in cells [16,17,18]. Moreover, levels of some ABC transporters such as ABCC1 and ABCG2 were reported to be upregulated by platinum compounds [50], thus we also monitored levels of ABCC1, ABCB1, and ABCG2.

**Figure 5:**
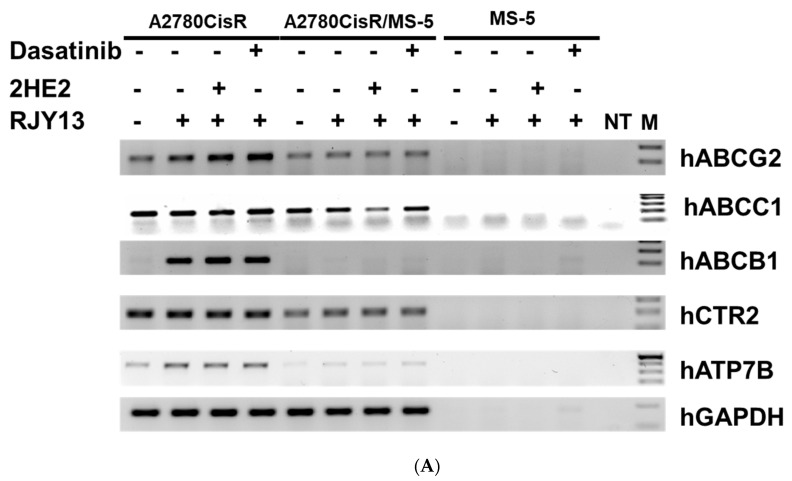

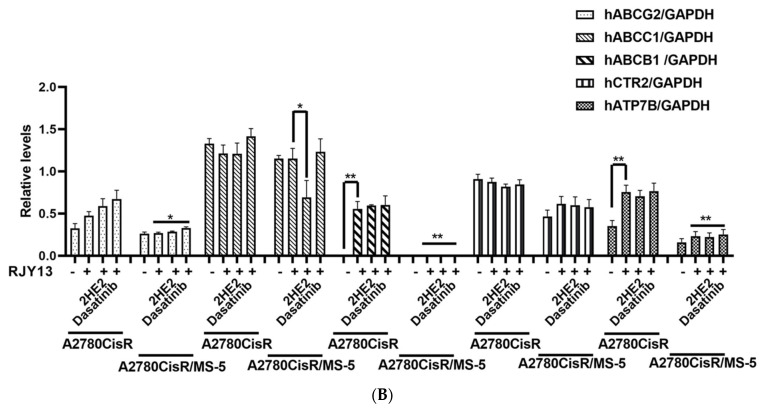
Effect of 2HE2 and dasatinib on expression levels of platinum transporters. (**A**) A2780CisR samples grown as monolayers or in direct co-culture with MS-5 were exposed to RJY13 (5 μM) for 24 h in the presence of 2HE2 (10 μM) and dasatinib (5 μM) as indicated. Total RNA was isolated and RT-PCR was performed as described in materials and methods. In this experiment, primers for hABCG2, hABCC1, hABCB1, hCTR2, hATP7B, hGAPDH, and mGAPDH were used as described in materials and methods. PCR products were separated at 1% agarose. Molecular weight DNA is also shown (M) and a sample without any temple (NT) served as negative control. (**B**) Transporter levels relative to levels of GAPDH were calculated and blotted. PCR products using mGAPDH are shown in Supplemental Figure S2. This experiment was repeated 3 times with similar results. * *p* < 0.05 and ** *p* < 0.01.

Semi-quantitative RT-PCR was used to monitor expression levels of some human copper and ABC transporters. Results demonstrated that the different amplicons were obtained only from human RNA, but not from murine RNA (Figure 5). This supports the species selectivity of the primers used. Results illustrated that the levels of a number of transporters, including hABCG2, hABCB1, and hATP7B, were upregulated in platinum-treated cells (Figure 5). In contrast, expression levels of hABCC1 and hCTR2 were not induced by platinum, and significant levels were present in the platinum-untreated samples. Moreover, levels of the transporters ATP7B, hABCB1, hCTR2, and hABCG2 were significantly downregulated in OC cells when co-cultured with MS-5, whereas levels of hABCC1 were only marginally downregulated in OC cells co-cultured with MS-5. Furthermore, levels of the different transporters were largely unchanged in cells treated with 2HE2 or dasatinib. In contrast, only 2HE2 marginally downregulated expression levels of hABCC1. These results confirmed that altered sensitivity to platinum is not related to the expression levels of copper or the ABC transporters examined in this study.

## 3. Discussion

Previously, we demonstrated that direct co-culture of OC with MSC conferred chemoresistance to platinum compounds caused by the blocking of ERK1/2 activation [15]. Moreover, combining platinum with fisetin restored platinum drug sensitivity to OC cells co-cultured with MSC by re-activating ERK1/2 activity [15]. Direct co-cultures facilitate physical contact between the different cell types, which allows for communication through their surface receptors. In this study, we examined the activity of additional modulators for their ability to restore platinum sensitivity in direct co-culture conditions of OC cells. Two modulators, 2-methoxyestradiol (2ME2) (Figure 1) and dasatinib (Figure 2) were successful in restoring partial platinum sensitivity to OC cells. Interestingly, the activity of 2-hydroxyestradiol (2HE2), a prodrug of 2ME2, was much more potent than the activity of 2ME2. 2ME2 is a non-active metabolite of estrogen that has been found to exhibit anticancer activities on several cancer cell lines [51,52]. 2HE2 exhibited very weak estrogenic activity [52] and was able to antagonize the estrogenic effect of estradiol [53]. 2HE2 is considered a prodrug of 2ME2 since the conversion of 2HE2 to 2ME2 is very efficient [25]. During the last few decades, studies have demonstrated that 2ME2 is a potent anti-angiogenesis inhibitor [22,23] because it downregulates hypoxia-inducible factor-1 (HIF-1) at the posttranscriptional level and inhibits HIF-1-induced transcriptional activation of VEGF expression [22]. Thus, we speculated that co-culture of OC with MSC might cause activation of HIF1α, which, in turn, would promote platinum chemoresistance [24,29]. Results demonstrated that no activation of HIF1α was observed in co-culture conditions (Figure 1B). Furthermore, exposure of OC co-culture to BAY 87-2243, a selective hypoxia-inducible factor-1 (HIF-1) inhibitor [30], did not restore platinum drug sensitivity to OC cells. Therefore, activity of 2ME2 or 2HE2 in restoring platinum drug sensitivity is not related to regulation of HIF1α activity. 

Our recent research demonstrated that quercetin and fisetin restored platinum sensitivity to OC cells co-cultured with MSC by promoting ERK1/2 activation [15]. Several studies have demonstrated that 2ME2 exposure promoted ERK1/2 activation in prostate cancer cells and in well-differentiated HK-1 cells, respectively [54,55]. Others, such as Lee et al. (2008) [56] have argued that 2ME2 induced a time-dependent inhibition of ERK1/2 and promoted activation of JNKs. Thus, we monitored ERK1/2 phosphorylation in OC cells exposed to 2HE2 or dasatinib. Our results demonstrated that re-sensitization of OC cells to platinum in co-cultures mediated by 2HE2 was not accompanied by activation of ERK1/2, in contrast to the platinum re-sensitization activity of dasatinib, which was accompanied by ERK1/2 activation (Figure 2). This finding supports the conclusion that platinum re-sensitization of OC might be ERK1/2-dependent for dasatinib and fisetin and ERK1/2-independent for 2HE2.

Interestingly, and in similar fashion to fisetin, a number of reports demonstrated the ability of dasatinib to stimulate ERK phosphorylation [32,33,57], whereas others demonstrated that dasatinib completely repressed nuclear ERK1/2 activity induced by HGF and EGF but not by bFGF [58]. Moreover, dasatinib enhanced doxorubicin-induced apoptosis in MCF-7/Adr cells by increasing the intracellular accumulation and inhibiting the efflux of doxorubicin into MCF-7/Adr cells. Activity of dasatinib in MCF-7/Adr cells is mediated, in part, by repressing ERK phosphorylation [59]. Here, dasatinib was active in stimulating ERK phosphorylation only in OC cells co-cultured with MS-5 and not in monolayers of OC cells (Figure 2), supporting potential alterations in signaling pathways in OC cells provoked by surface adhesion between OC and MS-5 cells.

To further investigate the involvement of ERK1/2 activation in mediating fisetin and dasatinib in restoring platinum sensitivity to OC cells, we utilized cobimetinib, an orally bioavailable small-molecule inhibitor of mitogen-activated protein kinase kinase 1 (MEK1), which also inhibits ERK phosphorylation [48]. Our results demonstrated that the combination with cobimetinib significantly reduces levels of cPARP as a result of exposure to RJY13 in the presence of dasatinib and fisetin, supporting the argument that the activity of dasatinib/fisetin in restoring platinum drug sensitivity to OC cells is dependent on ERK1/2 activity (Figure 3). In contrast, the activity of 2HE2 in restoring platinum drug sensitivity to OC was not affected by the presence of cobimetinib, providing further support for the argument that 2HE2 function in restoring platinum drug sensitivity to OC cells is ERK1/2 independent.

Reduced intracellular platinum accumulation has been consistently shown to correlate with resistance in tumors. Hence, to test a potential mechanism for the ability to mediate chemoresistance by direct co-culture, we measured platinum intracellular concentration in OC cells grown as monolayers compared with the concentration in direct co-culture with MSC. Results demonstrated a reduction of 35% in platinum intracellular concentration in OC cells grown in direct co-culture compared with OC cells grown as monolayers (Figure 4). We speculate that reduction in intracellular platinum concentration might be responsible, in part, for reduced sensitivity of OC cells to platinum simply because platinum activity is concentration dependent. Interestingly, modulators described in this study such as 2HE2, fisetin, and dasatinib were effective in increasing the platinum intracellular concentration in OC cells grown in direct co-culture with MSC (Figure 4B).

Platinum intracellular concentration is regulated by the copper transporters CTR1, CTR2, ATP7A, and ATP7B, which are essential for uptake and efflux in cells [16,17,18]. Previously, we demonstrated that uptake of our novel platinum compound RJY13 is independent of CTR1 [26]. Altered expression levels of genes involved in the uptake and efflux of platinum compounds are implicated in platinum drug resistance in cancer cells [16,17,18] and in cancer patients [16,59]. Thus, we focused on changes in levels of efflux transporters in OC cells grown in direct co-culture with MSC as a potential mechanism to reduce levels of platinum intracellular concentration. The overexpression of ABC transporters may lead to a resistance to conventional chemotherapeutics, radiotherapy, and targeted therapies [20]. Furthermore, low ABCB1 (P-glycoprotein) mRNA expression or truncation mutations are correlated with significantly longer survival in ovarian and other cancers [60]. Therefore, we monitored levels of copper transporters in OC cells that were grown as monolayers or in direct co-culture with MS-5 treated with RJY13 in combination with 2HE2, dasatinib, or fisetin. Results demonstrated that levels of a number of transporters, such as hABCG2, hABCB1, and hATP7B, were upregulated in platinum-treated OC cells (Figure 5). Our data are consistent with findings reported by other researchers demonstrating that cisplatin upregulates ABCB1 and ABCG2 gene expression regardless of the fact that cisplatin is not a substrate for the last two listed efflux transporters [61,62]. Moreover, gene expression of some transporters (ABCC1, ABCC2, ABCC3, and ABCB3) was significantly elevated in recurrent cancer lesions compared with benign or malignant ovarian tissue [63].

2ME2 was examined as a potential cancer sensitizer of cancer cells to standard therapy and found to be an effective radio-sensitizing agent in esophageal cancer (EC) cell lines [64] and also in human prostate cancer xenografts [65]. Moreover, dasatinib significantly increased the sensitivity of ABCB1-overexpressing cells, increased the intracellular accumulation, inhibited doxorubicin efflux, and significantly enhanced doxorubicin-induced apoptosis in ABCB1-overexpressing MCF-7 cells [59]. Furthermore, dasatinib also reversed ABCB1-mediated MDR by downregulating ABCB1 expression, which may be partly attributed to the inhibition of the ERK pathway [59].

The efflux transporters ABCB1 and ABCG2 have been demonstrated to interact with tyrosine kinase inhibitors (TKIs) such as dasatinib, and to reverse drug-resistance in human multidrug-resistant multiple myeloma cells mediated by Src inhibition [66]. Moreover, ABCB1 and ABCG2 are reported to be efflux transporters for dasatinib and other TKIs [67]. This cross-talk between dasatinib and ABC transporters might be involved in mediating dasatinib activity in restoring platinum sensitivity to OC cells.

In conclusion, our study yielded evidence that 2ME2/2HE2 and dasatinib are active in restoring platinum sensitivity to OC cells grown in direct co-culture with MSC cells. Our data have the potential to be translated into effective treatment for overcoming TME-mediated drug resistance in cancer patients. This possibility remains to be investigated.

## 4. Materials and Methods

Chemicals and assay kits: The chemicals 2-methoxyestradiol (2ME2), 2-hydroxyestradiol 2HE2 and GNF-5 were obtained from Cayman Chemical Company (Ann Arbor, MI, USA). The kinase inhibitors BAY-61-3606 and ZM39923 were obtained from Sigma Chemicals (Jerusalem, Israel). Tyrphostin AG-1296, PD98059, SB202190, and LY 294002 were obtained from Merck (Herzliya Pituach, Israel), Y-27632 from Thermo Fisher Scientific (Qiryat Shemona, Israel), and BAY 87-2243 from MedChemExpress (Monmouth Junction, NJ, USA). The flavonoid fisetin was obtained from Santa Cruz Biotechnology (Dallas, TX, USA). The FDA approved kinase inhibitors imatinib, ceritinib, gefitinib, ibrutinib, dasatinib and cobimetinib were obtained from CellChem Pharmaceuticals Inc. (Nepean, ON, Canada). 

The assay kit DC™ Protein Assay was obtained from Bio-Rad Laboratories (Hercules, CA, USA), Super Signal™ West Pico PLUS from Thermo Fisher Scientific (Qiryat Shemona, Israel), and Pathscan cleaved PARP (Asp214) sandwich ELISA kit from Cell Signaling Technology (Danvers, MA, USA).

Cell lines: Human platinum-sensitive and resistant OC cell lines A2780 and A2780CisR, respectively (obtained from the American Type Culture Collection [ATCC], Manassas, VA, USA), were cultured in RPMI 1640 complete medium supplemented with 10% (*w*/*v*) fetal bovine serum (Biological Industries, Beit-Haemek, Israel), 1% (*w*/*v*) L-glutamine, 100 units/mL penicillin, and 0.1 mg/mL streptomycin. The murine MSC lines MS-5 (also obtained from ATCC) were maintained under the same conditions. All cell lines were grown at 37 °C in a humidified atmosphere with 5% CO_2_.

In-vitro experiments: Experiments were carried out as previously described [15]. Briefly, human OC cell lines A2780 and A2780CisR were plated at 8 × 10^3^ cells/cm^2^ in 25 cm^2^ or 75 cm^2^ cell-culture flasks and incubated in culture medium. They were incubated as a monoculture or as a co-culture with MS-5, which were seeded at 4.2 × 10^3^ cells/cm^2^. After 24 h, cells were treated with platinum chemotherapy, consisting of 5 μM of our synthesized platinum (IV) prodrug (RJY13) [26] in combination with 10 μM of 2-methoxyestradiol (2ME2) and endogenous metabolite of 17β-estradiol, 2-hydroxyestradiol (2HE2) [46,47], imatinib [34], ceritinib [35], BAY-61-3606 [36], tyrphostin AG-1296 [37], PD98059 [38], SB202190 [39], gefitinib [40], Y-27632 [41], GNF-5 [42], ZM39923 [43], LY 294002 [44], and ibrutinib [45]. After additional 24 h incubation, the attached cells were collected using trypsin in RPMI medium. Cells were diluted 1:1 in trypan blue solution and counted using a hemocytometer), then centrifuged at 3000 rpm (1000× *g*) for 5 min and washed with cold phosphate buffered saline (PBS) twice to obtain cell pellets.

Trypan blue exclusion assay: Cells (2 × 10^5^/well) were plated in six-well plates. After 24 h, cells were treated with the specified agents. Solvent-treated samples were incubated with 0.1% (*w*/*v*) dimethyl sulfoxide. Cells were collected 72 h later, stained with 0.4% (*w*/*v*) trypan blue solution (1:1, *v*/*v*), and counted using a hemocytometer [68].

Immunoblotting: Protein analysis was performed by western blot protocol on an 8–12% acrylamide gel. Cell lysate samples were prepared for loading by adding lysis buffer (#9803 Cell Signaling Technology, Danvers, MA, USA) containing protease inhibitors (P8340 and P5726, Sigma, Roedermark, Germany) and phosphatase inhibitor (P-1517, AG Scientific, San Diego, CA, USA) to the cell pellets. After 30 min, samples were centrifuged and supernatants were tested for protein concentration using DC™ Protein Assay (Bio Rad, Hercules, CA, USA) and determining absorbance at 630 nm. Samples were lysed in lysis buffer, and 50–60 μg of protein per monoculture sample or 100–120 μg of protein per co-culture sample was loaded on the gel. Proteins were immunoblotted onto a nitrocellulose membrane (Schleicher & Schuell BioScience GmbH, Niedersachsen, Germany), which was then blocked with 5% skim milk TBS/T, and incubated with the following antibodies: anti-cleaved poly (ADP-ribose) polymerase (PARP) (Asp214) (D64E10, Cell Signaling Technology), anti-α-tubulin (sc-8035, Santa Cruz, TX, USA), and anti-phospho-ERK1/2 (Thr202/Tyr204) (D13.14.4E, Cell Signaling Technology) according to the manufacturers’ instructions. Secondary antibodies, HRP-linked anti-rabbit (#7074, Cell Signaling Technology) and anti-mouse (NB7539 Novus, Centennial, CO, USA) were used according to the manufacturers’ instructions. Chemiluminescence was performed with Super Signal™ West Pico PLUS Chemiluminescent Substrate (Thermo Fisher Scientific, Waltham, MA, USA) and imaged using an HP imager. Densitometry was performed with Image Quant v8.2 software.

Semi-quantitative RT-PCR: RT-PCR analysis was performed as previously described [69]. Briefly, total RNA was extracted from the cells using Tri Reagent (Sigma). Single-stranded cDNA was synthesized from the total RNA. A total of 1 μg RNA was preincubated with 1 μL oligo(dT)17 primer, and diethylpyrocarbonate (DEPC)-treated water was added to a total volume of 15 μL at 70 °C for 10 min; then the mixture was rapidly chilled on ice. To the annealed primer/template, 2 μL AMV RT 5× reaction buffer, 2 μL dNTP (25 mM), 28 units of RNasin ribonuclease inhibitor, 30 units of AMV RT, and DEPC-treated water were added to a final volume of 10 μL. The reaction was incubated at 42 °C for 60 min. The resulting cDNA was amplified with a PCR kit (Bioline, Taunton, MA, USA). The primer sets used for each gene are listed in Table 1. 

A total of 33–35 cycles of amplification were performed using the different primers with an initial incubation at 94 °C for 2 min and a final extension at 72 °C for 15 min; each cycle consisted of denaturation at 94 °C for 30 s, annealing at 55–60 °C for 30 s and an extension at 72 °C for 2:30 min. To ensure the use of equal amounts of cDNA from each sample in the PCR, the aliquots of the reverse transcription products were used with primers for the housekeeping gene GAPDH. A total 5 μL of the PCR products was analyzed by electrophoresis on a 1.5% agarose gel.

Cleaved cPARP ELISA: Cleaved cPARP assay was performed using a Pathscan cleaved PARP (Asp214) sandwich ELISA kit (Cell Signaling Technology, Danvers, MA, USA) according to the manufacturer’s instructions. The Human cleaved PARP ELISA Kit is a solid-phase sandwich Enzyme-Linked Immunosorbent Assay (ELISA) designed to detect and quantify the level of cleaved PARP in cell lysates. This assay utilizes an antibody that recognizes only the cleaved PARP protein from human origin. 

Measurements of platinum intracellular concentration: To monitor the platinum intracellular concentration of cisplatin (IV) prodrugs into OC cell lines, the experiment was carried out as previously described [49,70]. Briefly, cells were plated at 5 × 10^5^ cells/mL, and on the following day the compound RJY13 (5 μM) was added for 6 h. Cells were collected, washed four times with cold PBS and re-suspended in dH2O. Aliquots were removed for protein determination using the bicinchoninic acid (BCA) method according to the instructions of the manufacturer (Pierce, Rockford, IL, micro-well plate protocol). The remaining cell suspension was mineralized with 65% HNO3 and then completely dried at 120 °C. Dry platinum-containing material was dissolved in 2 mL of 2% HNO3. The platinum content was measured using an ICP-OES instrument (Agilent Technologies, Santa Clara, CA, USA). Platinum was measured at *m*/*z* 214. 

Statistical analysis: Statistical analysis was performed by Student’s *t*-test, with significant values at * *p* < 0.1, ** *p* < 0.01 and *** *p* < 0.001.

## Figures and Tables

**Table 1:** Primers used in this study.

	Forward Primer	Reverse Primer
hCTR2	5′-CTGCTGGCATGGCCCTTTCG-3′	5′-CTGTGGTGGGTTCTGCCAACAGG-3′
hATP7B	5′-ATC GGTTGTGTGCCTGCAACAGG-3′	5′-GGGTTAGTGCTTTGTAACCGCTCAAT-3′
hABCC1	5′-TGCCCTAGCCATCCTGAGAT-3′	5′-CCGGACAATCAACCCTGTGA-3′
hABCG2	5′-CAA CCATTGCATCTTGGCTG-3′	5′-CAAGGCCACGTGATTCTTCC-3′
hABCB1	5′-CAAATGCAAGAGGAGCAGCTTA-3′	5′-CCACTCTTCGAATAGCTGTCAA-3′
hGAPDH	5′-GAGTCAACGGATTTGGTCGT-3′	5′-GGTGCCATGGAATTTGCCAT-3′
mGAPDH	5′-CTGAGTATGTTGTGGAGTCTAC-3′	5′-CGTGGTTCACCATCACAAACATG-3′

## Data Availability

Data can be made available upon request.

## Supplementary Materials

The following Supplementary Materials can be downloaded online: Figure S1: cPARP levels in A2780CisR and MS-5 in RJY13 treated cells, and Figure S2: Effect of 2HE2 and dasatinib on expression levels of mGAPDH in A2780CisR co-culture with MS-5.

## Sample Availability

Samples of the compounds are not available from the authors.
