# Supplementary material for: 2-Hydroxyestradiol Overcomes Mesenchymal Stem Cells-Mediated Platinum Chemoresistance in Ovarian Cancer Cells in an ERK-Independent Fashion
Source: Molecules. 2022 Jan 26;27(3):804. doi: 10.3390/molecules27030804 (PMC8839885; doi:10.3390/molecules27030804)
Supplement: Supplementary file 1 [file molecules-27-00804-s001.zip › molecules-1550968-supplementary.pdf]

Supplemental material to Hazem Khamaisi et al., 2022

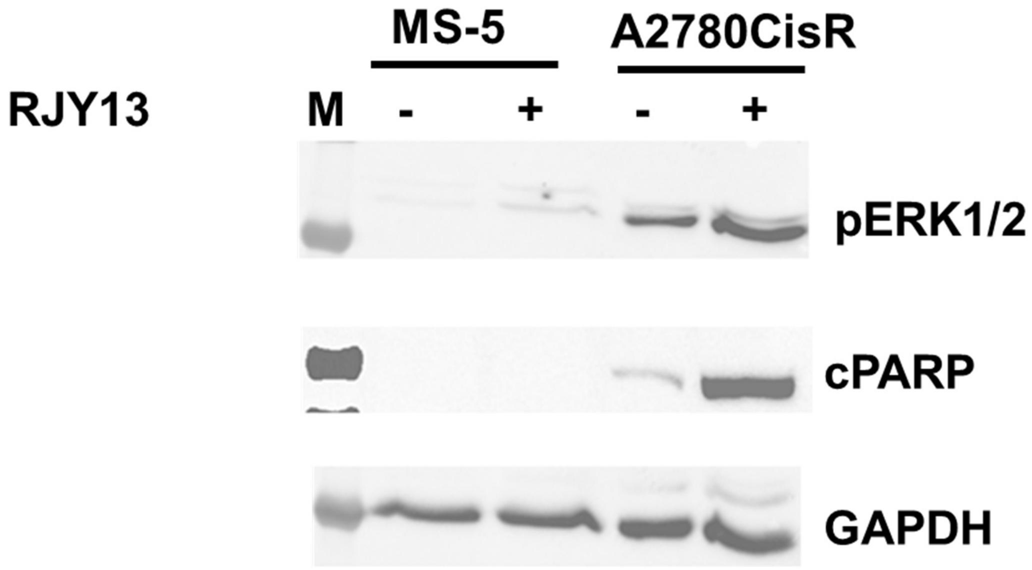

Figure S1. cPARP and pERK1/2 levels in A2780CisR and MS-5 in RJY13 treated cells

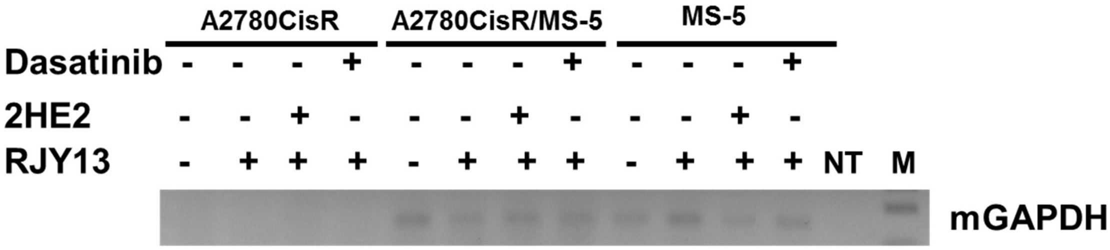

Figure S2. Effect of 2HE2 and dasatinib on the expression levels of mGAPDH in A2780CisR co-cultured with MS-5.
